# Uremic albumin blocks reverse cholesterol transfer: role of lysine modifications

**DOI:** 10.1186/1471-2210-11-S2-A32

**Published:** 2011-09-05

**Authors:** Veronika Binder, Michael Holzer, Dalia El-Gamal, Sabine Dirnberger, Gunther Marsche

**Affiliations:** 1 Institute of Experimental and Clinical Pharmacology, Medical University of Graz, 8010 Graz, Austria

## Background

Due to a lack of antioxidant enzymes in plasma, plasma proteins are preferential targets of oxidant injury. Reaction of chlorinated oxidants (e.g. hypochlorous acid), generated from activated neutrophils, with plasma albumin gives rise to formation of an oxidized protein fraction, termed advanced oxidation protein products (AOPPs). AOPP-albumin is a potent high-density lipoprotein (HDL) receptor antagonist, blocking HDL association and reverse cholesterol transport (RCT). However, it is not known whether structural alterations and/or an increase in negative charge (through oxidation of positively charged lysine residues on albumin) are required for high affinity binding to SR-BI.

## Methods and results

T-lymphocytes were incubated with AF 488-TFP-labelled HDL in the presence of different AOPP-albumin preparations (albumin oxidized *in vitro* by the myeloperoxidase product hypochlorous acid). HDL association to SR-BI was measured by flow cytometry. Our data show that already mild oxidation of albumin generates a high affinity ligand to SR-BI that effectively displaces HDL from the receptor. Oxidation/decomposition of lysine residues are required for binding of AOPP-albumin to SR-BI since masking lysine residues prior to oxidation as well as regeneration of lysine oxidation products completely averted binding. Interestingly, modification of albumin-located lysine residues with reactive carbonyls only moderately increased binding affinity of albumin to SR-BI.

## Conclusions

Structural alterations induced by lysine oxidation rather than an increased negative charge determine binding affinity of AOPP-albumin to SR-BI.

